# Regulating wave front dynamics from the strongly discrete to the continuum limit in magnetically driven colloidal systems

**DOI:** 10.1038/srep19932

**Published:** 2016-02-03

**Authors:** Fernando Martinez-Pedrero, Pietro Tierno, Tom H. Johansen, Arthur V. Straube

**Affiliations:** 1 Estructura i Constituents de la Matèria, Universitat de Barcelona, Av. Diagonal 647, 08028, Barcelona, Spain; 2 Institut de Nanociència i Nanotecnologia, Universitat de Barcelona, Barcelona, Spain; 3 Department of Physics, The University of Oslo, P.O. Box 1048 Blindern, 0316 Oslo, Norway; 4 Institute for Superconducting and Electronic Materials, University of Wollongong Innovation Campus, Squires Way, North Wollongong NSW 2500, Australia; 5 Department of Physics, Humboldt-Universität zu Berlin, Newtonstr. 15, D-12489 Berlin, Germany

## Abstract

The emergence of wave fronts in dissipative driven systems is a fascinating phenomenon which can be found in a broad range of physical and biological disciplines. Here we report the direct experimental observation of discrete fronts propagating along chains of paramagnetic colloidal particles, the latter propelled above a traveling wave potential generated by a structured magnetic substrate. We develop a rigorously reduced theoretical framework and describe the dynamics of the system in terms of a generalized one-dimensional dissipative Frenkel-Kontorova model. The front dynamics is explored in a wide range of field parameters close to and far from depinning, where the discrete and continuum limits apply. We show how symmetry breaking and finite size of chains are used to control the direction of front propagation, a universal feature relevant to different systems and important for real applications.

Examples of driven spatially discrete systems are widespread in both living and nonliving matter, ranging from signal propagation in biological cells^1^,^2^ to motion of interfaces^3^,^4^, charge density waves (CDWs)^5^,^6^,^7^,^8^, vortices in type-II superconductors^9^,^10^,^11^, frictional surfaces^12^,^13^. In general, a pinned system subjected to an external force may generate propagating fronts when reaching a threshold force. These fronts may induce depinning of the whole system, via transport of matter accompanied by energy dissipation. In discrete systems, emergence of propagating fronts under such conditions has received much theoretical attention in the past, mainly in relation to excitable cells^14^, burst waves in array of reaction sites^15^ and semiconductor superlattices^16^. In contrast, direct observations of wavefront dynamics in microscale systems have often been restricted to averaged quantities, such as current-voltage characteristics in CDWs^17^ and vortices in superconductors^18^, or dedicated surface imaging in chemical waves^19^.

Ensembles of interacting colloidal particles assembled above periodic optical^20^,^21^,^22^ or magnetic^23^,^24^ potentials represent simplified laboratory-scale model systems where the dynamics can be investigated in real time and space^25^. However, systems displaying well controlled fronts in ensembles of interacting colloidal particles are difficult to realize due to the strong damping of the dispersing medium, unless a pinning potential combined with an external force is used.

In this article, we explore the propagation of fronts along mobile chains assembled from interacting paramagnetic colloidal particles and driven above a magnetic structured film via a traveling potential landscape. In contrast to the magnetic chains propelling longitudinally with respect to the direction of motion^24^, here we employ a system where chains move perpendicular to their main axis. Although Brownian dynamics simulations are capable of replicating experimental observations, we develop a coarse grained analytically tractable description that admits a much deeper insight. We show that the complexity of the original system can be significantly reduced and it can be rigorously mapped to a generalized dissipative Frenkel-Kontorova (FK) model, allowing for a simple and accurate interpretation.

Being thoroughly studied in the conservative limit^26^, when the system becomes strictly nondissipative, the FK model is a cornerstone for understanding various nonlinear systems^27^,^28^, from coupled oscillators^29^ to discrete reaction-diffusion systems^30^, where many important questions remain open. Modifications of the FK model are also widely used in nanotribology^31^ to understand on a simplified ground frictional mechanisms occurring at the atomic scale^32^, or in sliding biological filaments^33^. In relation to the dissipative case, most efforts have been mainly theoretical and either focusing on the dynamics at^29^, or close to^30^,^34^, the depinning transition or those performed for continuum systems^8^,^35^. Our work presents an experimental realization of a dissipative FK system with emergent discrete fronts which can be generated and controlled by an applied external field. The dynamics of the system is systematically analyzed from the strongly discrete to the continuum limit by tuning the external field and consequently the coupling strength. Relevant for potential applications, we show that the finite system size allows us to polarize the emerging fronts via controlled symmetry breaking which results from uncompensated edge effects.

## Results

### Observing discrete fronts

**Figure 1:**
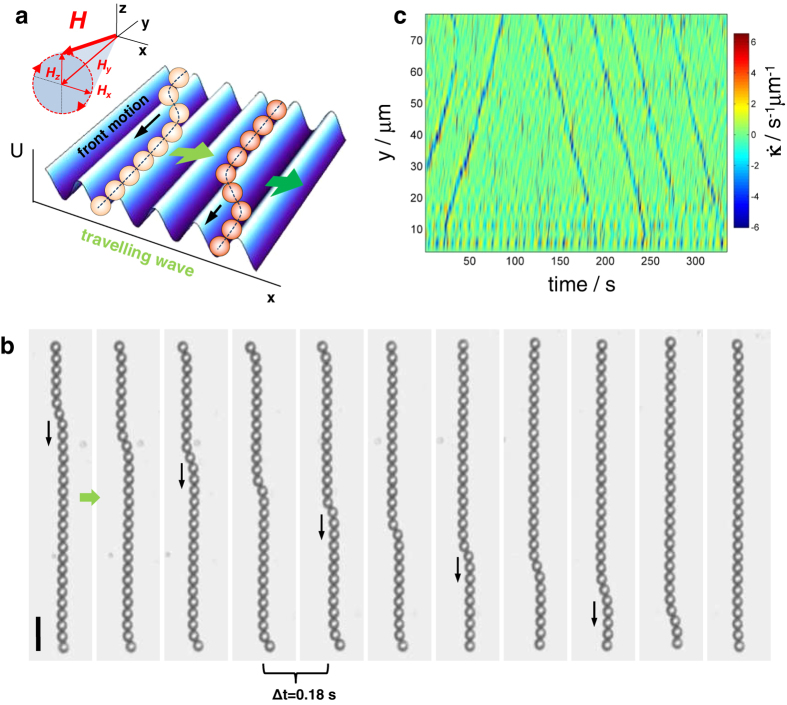
Propagation of fronts along propelling colloidal chains. (**a**) Schematic of a mobile chain of paramagnetic colloidal particles following a traveling periodic energy landscape, as realized by an external magnetic field precessing around the *y*-axis. The particles are held together by applying the static component *H*_*y*_ and the translation of the otherwise stationary periodic landscape along the *x* axis (green arrows) is prompted by the precession of the magnetic field. The chain is aligned along a valley of the landscape. Under the the combination of the drag forces and thermal fluctuations, a terminal particle jumps to the neighboring valley behind the chain, which triggers the front propagation along the chain (black arrows). (**b**) Sequence of images showing a front propagating downwards at a speed *v*_*f*_ = 26.7 μm s^−1^ along a chain composed of *N* = 28 paramagnetic colloidal particles (MovieS1 in Supporting Information). Field parameters are *H*_0_ = *H*_*y*_ = 1500 A m^−1^, *ω* = 37.7 rad s^−1^; time lapse between images is 0.18 s and the scale bar is 10 μm. (**c**) Space-time diagram of the chain bending rate 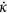, showing that fronts can propagate in both directions, upwards (along the *y* axis) and downwards (against the *y* axis).

We assemble and transport paramagnetic colloidal chains by using a bismuth substituted ferrite garnet film (FGF) characterized by a series of parallel ferromagnetic domains with alternating perpendicular magnetization and a spatial periodicity of *λ* = 2.5 μm (see Methods). The periodic arrangement of the nanoscale domain walls in the FGF film creates a one-dimensional (1D) sinusoidal potential landscape along the *x* direction, as shown in Fig. 1(a). Above this potential, we place paramagnetic microspheres of diameter *d* = 2.8 μ*m*, which are attracted by the stray field of the film **H**^sub^ and reside in minima of the energy landscape. In a magnetic field **H**, the particles acquire an induced dipole moment **m** = *υχ***H**, where *υ* = *πd*^3^/6 is the volume of particle and *χ* is the effective magnetic volume susceptibility of the particles. Application of an external alternating (ac) **H**^ac^ magnetic field rotating in the (*x, z*) plane,


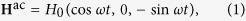


modulates the stray field of the FGF and causes the energy potential landscape to translate at a constant speed


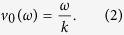


Here, *H*_0_ and *ω* are respectively the amplitude and angular frequency of the ac field and *k* = 2*π*/*λ* is the wave number of the landscape. At low enough frequency, the particles follow the running energy minima with an average translational speed 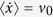.

To assemble the magnetic particles into a traveling chain aligned along the *y* axis, we add to **H**^ac^ a constant (dc) in-plane magnetic field, such that this field tilts the otherwise parallel magnetic moments along the *y* axis. As shown in ref. 36, the critical field above which the particles confined to the same minimum of the energy landscape experience net attractive interactions is:


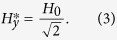


Note that this critical value can also be derived from the effective potential of mean force, see Eq. (19) in the Methods section. For the considered chain configuration, the effective potential describing dipolar interaction between a pair of particles in the chain is given by: 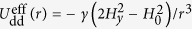, where *γ* = *μ*_0_(*υχ*)^2^/(8*π*) > 0, *μ*_0_ = 4*π* × 10^−7^ H m^−1^, and *r* is the distance between the particles. At the field given by Eq. (3) the dipolar force vanishes, separating the cases of repelling 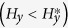 and attracting 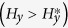 particles. Thus, *H*_*y*_ is used not only to assemble the particles in chains but also to control the chain stiffness, while **H**^ac^ is an independent means to induce their propulsion. A schematic showing the moving sinusoidal landscape with a chain of paramagnetic colloids is shown in Fig. 1(a).

A typical front is shown in Fig. 1(b), which is generated when the combination of the drag force and the thermal fluctuations displace a particle from its current dynamic equilibrium position in the propelling chain to the one that lags behind the chain by one spatial period *λ*. The front travels along the chain at an average speed *v*_*f*_ > *v*_0_. We quantify the chain transverse deformation by measuring the bending rate 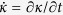, which describes the change in shape of the traveling chain^37^. Here, the curvature is given by *κ*(*s, t*) = |∂^2^***x***/∂*s*^2^| with *s* the arc length of the chain. As a consequence of the finite size of the chain, the fronts are typically excited at one of the two chain ends, since those particles only have one pulling neighbor and consequently are more susceptible to lose their phase. The spatial symmetry of the system with respect to *y* implies that fronts propagating upwards and downwards (against and along the *y* axis, respectively) are equally probable, as shown in Fig. 1(c).

### Coarse-grained model capable of front propagation

To obtain insight into the basic physics and quantify the dynamics of fronts, we apply a reduced one-dimensional (1D) model capable of front propagation along propelling chains. As shown in Methods, the complexity of full two-dimensional time-dependent system of a finite number *N* of magnetically interacting particles can be reduced. As a result of consistent coarse graining, the experimental system is cast into a generalized FK model with a “sine-Gordon” on-site potential *V*(*φ*) = *ω*_*c*_(1 − cos*φ*) − *ωφ*,


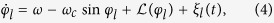



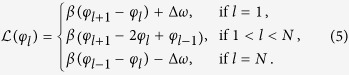


Note that the dynamics of particles with the coordinates *x*_*l*_ (*l* = 1, …, *N*) is formulated in the reference frame moving with the speed *v*_0_, Eq. (2), in terms of the phase variables, *φ*_*l*_(*t*) = −*k*(*x*_*l*_(*t*) − *v*_0_*t*).

The overdamped dynamics of phase in Eq. (4) is determined by the constant term *ω* caused by the external modulation, Eq. (1), the critical frequency *ω*_*c*_ that sets the amplitude of the sinusoidal landscape, a discrete linear coupling term originating from the dipolar interactions with the nearest neighbor particles, Eq. (5), and a stochastic term *ξ*_*l*_ effectively taking into account the presence of thermal fluctuations and possible structural disorder^38^. Here, *β* = *β*(*H*_0_, *H*_*y*_) is the coupling strength that has the dimension of frequency, Δ*ω* = Δ*ω*(*H*_*x*_, *H*_*y*_) is an effective frequency shift, and *ξ*_*l*_(*t*) is a Gaussian white noise with zero mean, 〈*ξ*_*l*_(*t*)〉 = 0, and covariance given by 〈*ξ*_*l*_(*t*)*ξ*_*l*′_(*t*′)〉 = 2*k*^2^*Dδ*_*ll*′_*δ*(*t* − *t*′) with *D* being the coefficient of Brownian diffusion (see Methods). For noninteracting particles (*γ* = 0), the coupling term vanishes, 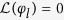, Eq. (4) reduces to the generic stochastic Adler equation (for the discussion of its properties, see refs 39,40),


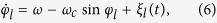


which admits a stable phase-locked solution Φ(*ω*) = arcsin(*ω*/*ω*_*c*_) for *ω* < *ω*_*c*_ and a phase-drift solution, *φ*_*l*_ = *φ*_*l*_(*t*), with the deterministic mean speed 
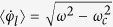
 for *ω* > *ω*_*c*_.

We note that the invariance of Eqs. (4) and (5) with respect to the transformation *φ*_*l*_ → *φ*_*l*_ + 2*π* makes it effectively bistable with the stable equilibria *φ*_−_ = Φ and *φ*_+_ = Φ + 2*π*. As a result, for *β* > 0, the model given by Eqs. (4) and (5) admits monotonic discrete front solutions^8^,^34^


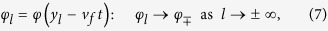



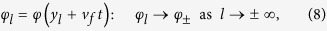


with a front speed *v*_*f*_(*ω, β*) > 0, describing the fronts traveling along and against the *y* axis, respectively.

It is also important to note that the parameter Δ*ω*, which enters only the governing equations for the terminal particles, introduces asymmetric frequency shifts, *ω* → *ω* ± Δ*ω*, cf. Eqs. (4) and (5) for *l* = 1 and *l* = *N*. In the partial case of Δ*ω* = 0, Eqs. (4) and (5) are invariant under the transformation *y* → −*y*, and the fronts traveling in opposite directions, along and against the *y* axis, remain equally probable. This property of the model reflects the experimental observation shown by Fig. 1(c). The case Δ*ω* ≠ 0, however, breaks this symmetry and front solutions in Eqs. (7) and (8) are no longer equally probable. Depending on the sign of the parameter Δ*ω*, one (or the opposite one) direction of front propagation becomes preferable. Both these situations are discussed below.

### Dynamic state diagram. Discrete fronts as a result of depinning of flexible chains

**Figure 2:**
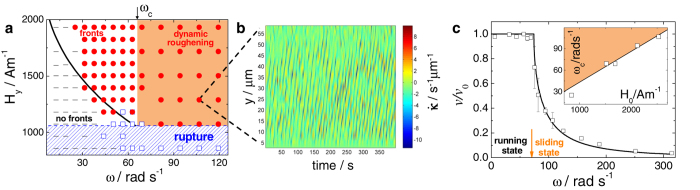
Existence of fronts and dynamics of individual colloidal particles. (**a**) State diagram in the (*ω, H*_*y*_) plane denoting regions with no fronts (black segments), propagating fronts (red circles), dynamic roughening (shaded area) and chain rupture (blue squares) below the blue dashed line 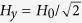 with *H*_0_ = 1500 A m^−1^. The depinning transition, *ω*_*d*_(*H*_*y*_) (solid line), is calculated by numerically solving Eqs. (4) and (5); The asymptotic behavior of *ω*_*d*_(*H*_*y*_) at small and large values of *H*_*y*_ is in accordance with Eqs. (9) and (10), respectively; recall that *β* = *β*(*H*_*y*_), see Eq. (23) in Methods. (**b**) Space-time diagram of 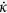 of a chain showing dynamic roughening occurring in the sliding state, see MovieS2 in the Supporting Information. (**c**) Normalized velocity of a single particle *v*/*v*_0_ versus angular frequency *ω* for *H*_0_ = 1500 A m^−1^ showing the transition from the running to sliding states separated by a critical frequency *ω*_*c*_. In the inset we show the critical frequency versus *H*_0_.

We now analyze the symmetric case, Δ*ω*(*H*_*x*_ = 0) = 0, when the asymmetry in the behavior of the terminal particles described by Eqs. (4) and (5) for *l* = 1 and *l* = *N* disappears. For this reason, properties of front propagation exhibited by finite chains composed of few tens of particles can be drawn equally well from the model of an infinite chain. In Fig. 2(a), we show a state diagram illustrating the various dynamic regimes and types of chain deformation experimentally observed in the (*ω, H*_*y*_) plane. We first note that the propelling chain is stable for positive coupling strengths *β* > 0, which corresponds to large enough fields, 
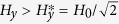
, as follows from Eq. (3) and Eq. (23) in the Methods section. For smaller fields 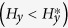, the coupling strength is negative, *β* < 0, and the chain breaks up. The critical line 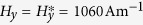 predicted by the model for *H*_0_ = 1500 A m^−1^ is in agreement with the experimental observations, Fig. 2(a). Above this line, for *ω* ∈ [25.1, 64.7]rad s^−1^, we observe chains running with a net speed of *v* ≈ *v*_0_ accompanied by fronts propagating along the chains, where *v*_0_ is given by Eq. (2). For frequencies 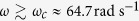, the chain propulsion slows down and stable fronts are no longer observed.

This behavior is explained by a global instability of Eq. (4) at *ω* = *ω*_*c*_(*H*_0_), at which the stable equilibria *φ*_±_(existing for *ω* < *ω*_*c*_) disappear and the phase starts to drift. As described by Eq. (6), a deterministic chain of noninteracting particles either locks to the landscape and runs together with it uniformly, with the constant speed *v*_0_ (*ω* < *ω*_*c*_), or decouples from the landscape and slides across it asynchronously with a lower net speed, 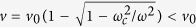 (*ω* > *ω*_*c*_), see Fig. 2(c). The extinction of stable equilibria *φ*_±_ prohibits front solutions. We confirm this expectation by observing beyond *ω*_*c*_ multiple bending fluctuations typical of dynamic roughening phenomena^41^, as shown in the space time plot of 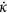 in Fig. 2(b).

From Fig. 2(a) it becomes clear that there is also a lower bound for the existence of fronts. It is set by the dependence *ω* = *ω*_*d*_(*β*) < *ω*_*c*_, which represents the depinning transition caused by the discretness of the system. At low frequencies, *ω* < *ω*_*d*_, inhomogeneous solutions with front-like profiles remain “pinned” and fail to propagate. The fronts depin and start to propagate for *ω* > *ω*_*d*_. The critical dependence *ω*_*d*_ = *ω*_*d*_(*β*(*H*_*y*_)) is evaluated numerically by solving the FK model, Eqs. (4) and (5).

In the strongly discrete limit, when front profiles are sharp, *ω*_*d*_ is largest and can be evaluated similarly to the approach applied in ref. 30,


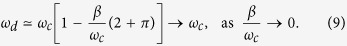


In the continuum limit^29^, the dependence is known to decay exponentially with the coupling strength,


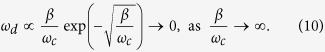


In this limiting case, fronts are smooth and the discrete FK model, Eqs. (4) and (5) applied to an infinite chain, can be approximated by a conventional continuum reaction-diffusion equation. The discrete coupling term, *β*(*φ*_*l*+1_ − 2*φ*_*l*_ + *φ*_*l*−1_), is replaced by a 1D continuum diffusion term, *βd*^2^∂_*yy*_*φ*.

### Transition from the strongly discrete to continuum limit for the front speed

**Figure 3:**
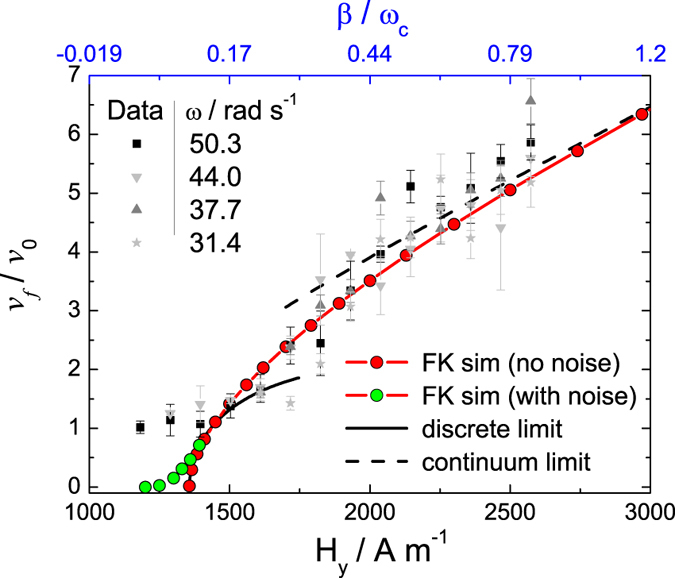
Magnetic control of front speed switching from the discrete to continuum limits. Normalized front velocity *v*_*f*_/*v*_0_ as functions of the magnetic field *H*_*y*_ (bottom axis) and the normalized coupling strength *β*/*ω*_*c*_ (top axis). Scattered symbols denote experimental data for different *ω* (*H*_0_ = 1500 A m^−1^). Continuous (red) lines with filled red and green circles are results of respectively deterministic (*D* = 0) and stochastic (effective diffusion coefficient 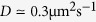) simulations of Eqs. (4) and (5) for *N* = 20 particles. The value of *D* in the stochastic simulation effectively accounts for both thermal fluctuations and structural disorder, leading to rounding of the transition close to the depinning point and converging to the deterministic result away from it. Black continuous and dashed lines represent the predictions in the discrete, Eq. (11), and continuum, Eq. (12), limits, respectively.

In order to characterize the front dynamics we perform a series of experiments by measuring the front velocity *v*_*f*_ versus the field amplitude *H*_*y*_ for different driving frequencies *ω*. Figure 3 shows the growth of the normalized front speed *v*_*f*_/*v*_0_ with the increase in *H*_*y*_, which demonstrates a smooth transition from the strongly discrete to continuum limit predictions. Indeed, close to the depinning transition at *H*_*y*_ ≈ 1400 A m^−1^, the front speed can be evaluated as


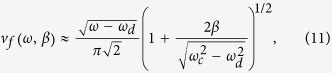


as analytically derived in ref. 34. Note that because *ω*_*d*_ is a function of *β*, cf. Eq. (9), the dependence of *v*_*f*_ on *β* deviates from a simple power law. In the continuum reaction-diffusion formalism we obtain an estimate,


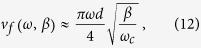


which is consistent with the scaling 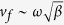 reported previously^29^. To derive analytic prediction (12), we assumed that the front profile does not significantly deviate from the stationary kink solution valid at *ω* = 0, which is justified in the continuum approximation and is a posteriori confirmed by numerical simulations in the continuum limit.

We emphasize that neither of the asymptotic predictions, Eqs. (11) and (12), are uniformly valid in the whole range of *H*_*y*_, when compared with the results of simulations of Eqs. (4) and (5), shown as filled circles in Fig. 3. The numerical data are in good agreement with the experimental ones in the whole range of *H*_*y*_ for *γ*/*ζ* = 75 μm^7^mA^−2^s^−1^, used as a fit parameter with *ζ* being the coefficient of viscous friction. The only discrepancy occurs close to the depinning point, where the experimental data deviate from the theoretical prediction.

On one hand, this distinction can be attributed to the presence of thermal fluctuations and structural disorder. As known from the literature^42^,^43^, these factors accounted as effective thermal noise result in rounding of the transition in the vicinity of depinning. As confirmed by our simulations (see Fig. 3), while the purely deterministic limit displays a sharp transition (red circles), the presence of thermal noise leads to its softening (green circles). On the other hand, a reliable estimate for the front speed close to the depinning point, where front speeds are small, requires accumulation of large enough statistics. These amounts of data can be obtained within the framework of the numerical model but are not always available in the experiment.

However, because *β* = *β*(*H*_*y*_), see Eq. (23) in the Methods section, by uniformly changing *H*_*y*_, our experimental system allows us to explore systematically the whole range of *β*, from strongly discrete, *β* ≪ *ω*_*c*_, to nearly continuum limits. From the comparison of the results of the full FK model, Eqs. (4) and (5), with the continuum-limit prediction Eq. (12) in Fig. 3, we conclude that the continuous limit sets already at 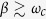.

### Controlling the direction of front propagation

**Figure 4:**
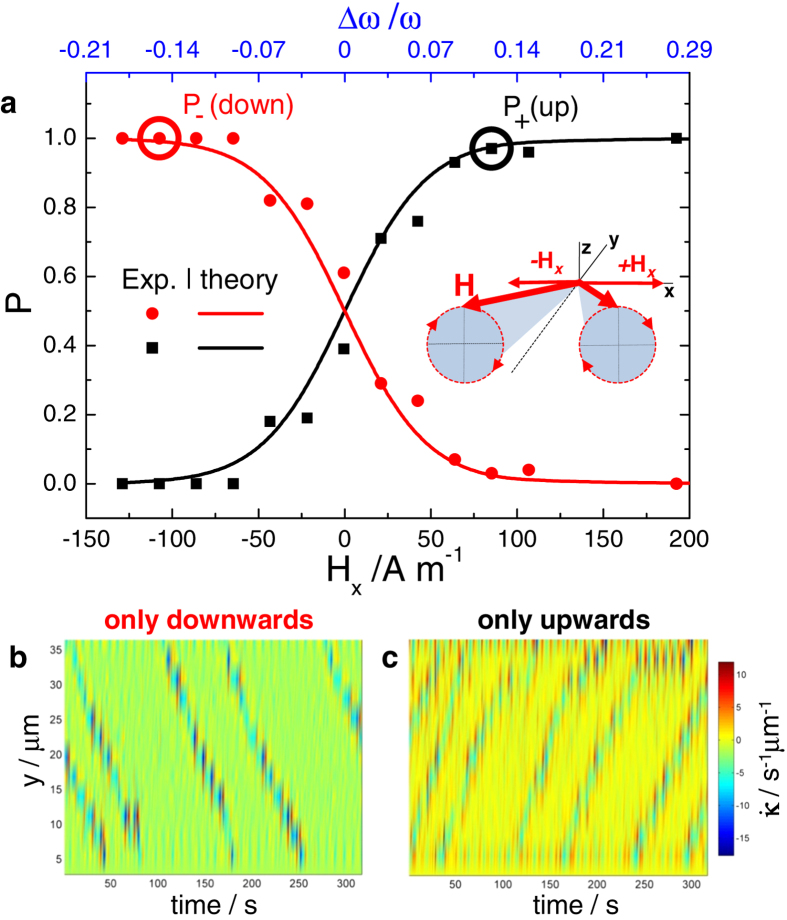
Magnetic control of the direction of front propagation. (**a**) Probabilities to observe fronts propagating upwards, *P*_ + _ (black), and downwards, *P*_−_ (red), as functions of constant field *H*_*x*_ (bottom axis) and Δ*ω*/*ω* (top axis) for *H*_0_ = *H*_*y*_ = 1500 A m^−1^, *ω* = 37.7 rad s^−1^. Points correspond to experimental data, and lines are fits according to the theoretical model, Eq. (13), performed as described in the text. (**b**,**c**) Space-time diagrams of the bending rate 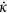 showing the polarization of fronts moving either downwards (**b**) or upwards (**c**). Experimental conditions correspond to the two circles in panel (**a**).

Finally, we demonstrate how by adding a small constant component *H*_*x*_ to the external field such that |*H*_*x*_| ≪ *H*_0_, we can control the direction of front propagation. In Fig. 4, we plot the probabilities to observe an upwards, *P*_+_, or downwards, *P*_−_, propagating front versus *H*_*x*_. The physical mechanism underlying this front polarization effect is based on an interplay between the finite size of chains, which implies broken spatial symmetry of the system for *H*_*x*_ ≠ 0 for terminal particles, and thermal noise.

We recall that front propagation is typically triggered by a terminal particle in the chain, as soon as the terminal particle overcomes a potential barrier Δ*V* needed to undergo the transition *φ*_−_ → *φ*_+_. As follows from Eqs. (4) and (5), for the perfectly symmetric case, *H*_*x*_ = 0, the stable equilibria are *φ*_−_(*ω*) = Φ(*ω*) and *φ*_+_(*ω*) = Φ(*ω*) + 2*π* with Φ(*ω*) = arcsin(*ω*/*ω*_*c*_), and the corresponding barrier Δ*V*(*ω*) = *πω* − 2*ω*Φ(*ω*)− 2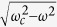. The symmetry of the model is broken for the terminal particles via the term |Δ*ω*(*H*_*x*_)| ≪ *ω* with Δ*ω* ∝ *H*_*x*_, see Eq. (23) in Methods section. Therefore, the equilibria positions for the terminal particles are slightly shifted from *φ*_±_(*ω*) for *H*_*x*_ = 0 to *φ*_±_(*ω* ± Δ*ω*) for *H*_*x*_ ≠ 0. Note that the equilibria positions of the inner particles remain unaffected, which particularly indicates that the mechanism is essentially independent of the chain length, ensuring its universality. As a result, the barrier Δ*V*(*ω* ± Δ*ω*) which the terminal particle has to overcome in the case *H*_*x*_ ≠ 0 decreases for ±Δ*ω* > 0 and increases for ±Δ*ω* < 0, respectively.

In the presence of thermal fluctuations, the associated Kramers’ escape rates, *r*_±_(Δ*ω*) ∝ exp[−Δ*V*(*ω* ± Δ*ω*)/(*k*^2^*D*)] lead us to the probabilities *P*_±_ = *r*_±_/(*r*_+_ + *r*_−_). Their approximate evaluation yields the following expressions:


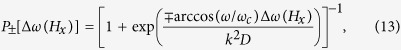


which are used to fit the experimental data (scattered points) in Fig. 4. Equation (13) can be considered as a function of the *H*_*x*_ field, 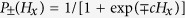, where *c* is a fit parameter. A good agreement between this analytic prediction and the experimental data is achieved at *c* = 0.04 mA^−1^. A similar dependence in terms of *β* can be obtained by using the relation between *β* and *H*_*x*_ see Eq. (23) in the Methods section.

As follows from the prediction for *P*_±_, Eq. (13), the fronts propagating upwards and downwards are equally probable at *H*_*x*_ = 0, *P*_+_ = *P*_−_ = 1/2, which is well seen from Fig. 4(a). This finding is expected from the discussed symmetry of the model, since at *H*_*x*_ = 0 the frequency shift Δ*ω* = 0 and Eqs. (4) and (5) become invariant with respect to the transformation *y* → −*y*. We note that exponential dependence of *P*_±_ on *H*_*x*_ ensures that already relatively small values of *H*_*x*_ ≠ 0 allow us to polarize the fronts. Indeed, at |*H*_*x*_| = 100 A m^−1^, which is much smaller than the fields *H*_0_ = *H*_*y*_ = 1500 A m^−1^, we can have polarized fronts propagating either downwards (*P*_−_ ≈ 1, *P*_+_ ≈ 0, *H*_*x*_ < 0) or upwards (*P*_−_ ≈ 0, *P*_+_ ≈ 1, *H*_*x*_ > 0), depending on the sign of *H*_*x*_, see Fig. 4(b,c).

## Conclusions

We have presented an experimental system showing discrete fronts which propagate along chains of paramagnetic colloidal particles held together and driven by an external magnetic field. We develop a reduced analytically tractable model which exquisitely matches and explains the observed features in a wide range of field strengths ranging from the strongly discrete to continuum limits. The finite size of chains is used to polarize the emerging front via a symmetry breaking mechanism. We note that the actual number of particles in the chain is unimportant and the mechanism is effectively universal, being valid for both short and long chains. The ability to control the front dynamics, including its propagation direction, in driven colloidal systems is appealing for potential applications such as e.g. transport and precise positioning of functionalized cargos above moving fronts or colloidal fractionation in nanofluidic sieving processes^36^. Finally, the generic form of the developed model presents a firm implication for a greater variety of systems exhibiting similar phenomena. Particular details of our experimental system present no restrictions for our findings, which can expectedly be extended to other nonlinear systems in biological and condensed matter contexts.

## Methods

### Experimental colloidal system

We used aqueous suspension of monodisperse paramagnetic colloidal particles (Dynabeads M-270, Dynal) of diameter *d* = 2.8 μm and effective magnetic volume susceptibility *χ* = 0.4. The particles are paramagnetic due to the uniform doping (20% by weight) with iron-oxide grains. The stripe patterned ferrite garnet film (FGF) of wavelength *λ* = 2.5 μm was grown by dipping liquid phase epitaxy on a gadolinium gallium garnet substrate^44^. The particles were diluted in highly deionized water and deposited above the FGF surface. We prevented particle adhesion to the FGF substrate by coating the latter with a 1 μm thick layer of a photoresist (AZ-1512 Microchem, Newton, MA) via standard spin coating and backing procedures.

The applied magnetic field was provided via custom-made Helmholtz coils perpendicular to each other. The coils were connected to two independent bipolar amplifiers (Kepco BOP 20-10M, KEPCO) controlled with a wave generator (TGA1244, TTi). To visualize the particle dynamics we used an upright optical microscope (Eclipse Ni, Nikon) which was equipped with a 100 × 1.3 NA oil immersion objective and a CCD camera (Balser Scout scA640-74fc) working at 75 frames per second. A total field of view of 145 × 109 μm^2^ was obtained by adding before to the optical path a 0.45 × TV adapter.

### General theoretical framework

We start by considering an ensemble of *N* interacting colloidal particles placed above the FGF at a fixed elevation *z* and in-plane positions **r**_*l*_ = (*x*_*l*_, *y*_*l*_) with *l* = 1, …, *N*. The dynamics of particles can be well described by two-dimensional (2D) overdamped Langevin equations


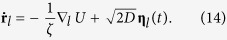


Here *ζ* is the coefficient of viscous friction, *D* of Brownian diffusion, and **η**_*l*_ = (*η*_*xl*_, *η*_*yl*_) is a Gaussian white noise with zero mean, 〈*η*_*αl*_(*t*)〉 = 0, and unit covariance matrix, 〈*η*_*αl*_(*t*)*η*_*α*′*l*′_(*t*′)〉 = *δ*_*αα*′_*δ*_*ll*′_*δ*(*t* − *t*′), where *α*,*α*′ ∈ {*x, y*}.

The total magnetic energy of the ensemble of induced dipoles is given by


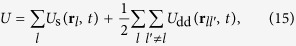


where


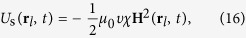



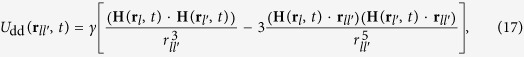


describe the individual interaction of particles with the magnetic field above the substrate and pairwise dipolar interactions, respectively. Here *γ* = *μ*_0_(*υχ*)^2^/(8*π*), **r**_*ll*′_ = **r**_*l*_ − **r**_*l*′_ are the relative coordinates, and *r*_*ll*′_ = |**r**_*ll*′_| the interparticle distances.

The total field above the FGF is evaluated as **H** = **H**^ac^ + **H**^dc^ + **H**^sub^ with the external time alternating field **H**^ac^ = (cos*ωt*, 0, −sin*ωt*), the external constant field **H**^dc^ = (*H*_*x*_, *H*_*y*_, 0), and the field of substrate **H**^sub^ = (4*M*_*s*_/*π*)e^−*kz*^(cos*kx*, 0, −sin*kx*)^45^. Here, *ω* is the angular frequency of modulation, *M*_*s*_ is the saturation magnetization and *k* = 2*π*/*λ* the wave number of the substrate. The *H*_*x*_ field is assumed to be small, |*H*_*x*_| ≪ *H*_0_, and is used to control the direction of the front propagation.

### Derivation of the reduced theoretical model

To derive an efficient rigorously reduced one-dimensional (1D) description in terms of a generalized Frenkel-Kontorova (FK) model, we perform two consecutive steps reducing the complexity of the full 2D time-dependent model, Eqs. (14)-(17), 32, 33, 34.

First, by integrating out the “fast” oscillatory timescale *τ* = *ω*^−1^, we focus on the slow dynamics at times 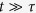
46,47, to arrive at effective potentials of mean force


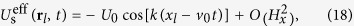



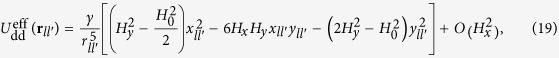


where *U*_0_ = (4/*π*)*μ*_0_*υχM*_*s*_*H*_0_e^−*kz*^, *v*_0_(*ω*) = *ω*/*k*, and we have retained only the leading linear contributions in *H*_*x*_ and neglected the smaller higher order terms 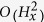. Note that while obtaining Eq. (19), we have assumed that the dipolar interaction is mainly caused by the externally applied field and therefore **H** ≈ **H**^ac^ + **H**^dc^, as particularly confirmed earlier^36^,^47^. We also note that the time dependence enters Eq. (18) via the combination ∝(*x*_*l*_ − *v*_0_*t*) typical of the wave propagation, reflecting the translation of the spatially periodic energy landscape with the speed *v*_0_. Thus, expressions (18) and (19) are time independent (and the corresponding slow-timescale equations of motion are fully autonomous) in the co-moving reference frame.

Next, assuming a ground state in the form of chain with *y*_*l*_ = *ld* + const, we consider Eqs. (14) and (15) with *U*_*s*_(**r**_*l*_, *t*) and *U*_*dd*_(**r**_*ll*′_, *t*) replaced by the effective potentials (18) and (19), which leads to 1D equations of motion


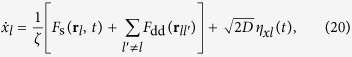


with the forces 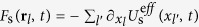 and 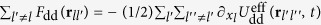. While the evaluation of the force due to individual interaction with the field of substrate yields a simple expression


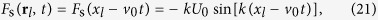


the terms describing the dipolar interaction are cumbersome. Therefore, to arrive at an analytically tractable model, the dipolar force is evaluated approximately by linearizing it with respect to the coordinates *x*_*ll*′_ and retaining the interactions with the nearest neighbors only. Note that although dipolar forces are long ranged and formally require to account for further neighbors^48^, the nearest neighbor approximation is often successfully applied to simplify the theoretical analysis^41^ and is known to work particularly well for systems of paramagnetic colloidal particles coupled via dipolar interactions both in and out of equilibrium^49^. As a result, for the magnetic force exerted on particle *l* by particles *l* ± 1, we obtain


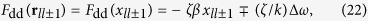


where the following parameters are introduced:


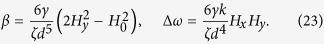


Thus, the dynamics of the magnetic chain is reduced to 1D equations of motion


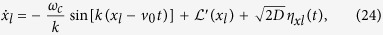


where *ω*_*c*_ = *k*^2^*U*_0_/*ζ*= 8*μ*_0_*υχkM*_*s*_*H*_0_/(*ζλ*)e^−*kz*^ is a critical frequency and


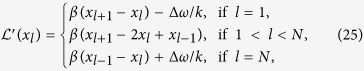


is the linear coupling term caused by dipolar interactions with the nearest neighbors. Finally, proceeding to the reference frame moving with the speed *v*_0_, introducing a phase variable *φ*_*l*_(*t*) = −*k*(*x*_*l*_(*t*) − *v*_0_*t*), and taking into account that 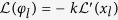 in Eqs. (24) and (25), we arrive at our generalized FK model given by Eqs. (4) and (5), where *β* stands for the coupling strength, Δ*ω* is an effective frequency shift, and the stochastic term is rescaled as 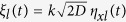.

## Additional Information

**How to cite this article**: Martínez-Pedrero, F. *et al*. Regulating wave front dynamics from the strongly discrete to the continuum limit in magnetically driven colloidal systems. *Sci. Rep*. **6**, 19932; doi: 10.1038/srep19932 (2016).

## Supplementary Material

Supplementary Movie S1

Supplementary Movie S2

Supplementary Information
