# Supplementary material for: Regulating wave front dynamics from the strongly discrete to the continuum limit in magnetically driven colloidal systems
Source: Sci Rep. 2016 Feb 3;6:19932. doi: 10.1038/srep19932 (PMC4738245; doi:10.1038/srep19932)
Supplement: Supplementary Information [file srep19932-s3.doc]

Description of the supporting videos for the article:

**Regulating wave front dynamics from the strongly discrete to the continuum limit in magnetically driven colloidal systems**

By Fernando Martinez-Pedrero, Pietro Tierno, Tom H. Johansen, and Arthur V. Straube

With the article there are 2 movies in support of Figs. 1 and 3.

**MovieS1**.(AVI): The dynamics of a chain of N = 30 particles transported above the FGF surface upon application of an external precessing magnetic field with amplitudes *H_0_ = H_y_ = 1500 Am^-1^* and angular frequency ω = 37.7 rad s^-1^. The movie shows the emergence of fronts traveling along the chain in both directions, upwards and downwards.

**MovieS2**.(AVI): Dynamics of a driven chain of N = 22 paramagnetic colloidal particles transported above the FGF surface upon application of an external precessing magnetic field having amplitudes *H_0_ = 1500 Am^-1^* and *H_0_ = 1300 Am^-1^* and angular frequency ω = 106.8 rad s^-1^. The movie shows the dynamic roughening of the chain, which results at the frequency higher than the critical one ω_c_ = 62.8 rad s^-1^.
